# Rationally Improved Surface Charge Density of Triboelectric Nanogenerator with TiO_2_‐MXene/Polystyrene Nanofiber Charge Trapping Layer for Biomechanical Sensing and Wound Healing Application

**DOI:** 10.1002/advs.202404019

**Published:** 2024-07-09

**Authors:** Manikandan Venkatesan, Jayashree Chandrasekar, Yung‐Chi Hsu, Ting‐Wang Sun, Po‐Yu Li, Xuan‐Ting King, Ming‐An Chung, Ren‐Jei Chung, Wen‐Ya Lee, Ye Zhou, Ja‐Hon Lin, Chi‐Ching Kuo

**Affiliations:** ^1^ Institute of Organic and Polymeric Materials National Taipei University of Technology Taipei 10608 Taiwan; ^2^ Department of Electronic Engineering National Taipei University of Technology Taipei 10608 Taiwan; ^3^ Department of Chemical Engineering and Biotechnology National Taipei University of Technology Taipei 10608 Taiwan; ^4^ Institute for Advanced Study Shenzhen University Shenzhen 518060 P. R. China; ^5^ Department of Electro‐Optical Engineering National Taipei University of Technology Taipei 106 Taiwan; ^6^ Advanced Research Center for Green Materials Science and Technology National Taiwan University Taipei 10617 Taiwan

**Keywords:** charge trapping, electrical stimulation, electrospun nanofiber, oxidized MXene, triboelectric nanogenerator, wound healing

## Abstract

Triboelectric nanogenerators (TENGs) have become reliable green energy harvesters by converting biomechanical motions into electricity. However, the inevitable charge leakage and poor electric field (EF) of conventional TENG result in inferior tribo‐charge density on the active layer. In this paper, TiO_2_‐MXene incorporated polystyrene (PS) nanofiber membrane (PTMx NFM) charge trapping interlayer is introduced into single electrode mode TENG (S‐TENG) to prevent electron loss at the electrode interface. Surprisingly, this charge‐trapping mechanism augments the surface charge density and electric output performance of TENGs. Polyvinylidene difluoride (PVDF) mixed polyurethane (PU) NFM is used as tribo‐active layer, which improves the crystallinity and mechanical property of PVDF to prevent delamination during long cycle tests. Herein, the effect of this double‐layer capacitive model is explained experimentally and theoretically. With optimization of the PTMx interlayer thickness, S‐TENG exhibits a maximum open‐circuit voltage of (280 V), short‐circuit current of (20 µA) transfer charge of (120 nC), and power density of (25.2 µW cm^−2^). Then, this energy is utilized to charge electrical appliances. In addition, the influence of AC/DC EF simulation in wound healing management (vitro L929 cell migration, vivo tissue regeneration) is also investigated by changing the polarity of trans‐epithelial potential (TEP) distribution in the wounded area.

## Graphical abstract

Surface charge density of single electrode mode triboelectric nanogenerators (S‐TENG) is boosted by integrating TiO_2_‐MXene doped polystyrene nanofiber membrane (PTMx) as a capacitive interlayer. This enhanced charge trapping reduces energy loss and improves TENG performance. The generated output energy is utilized for accelerating wound healing, highlighting its potential impact in sensors and medicinal research due to its flexibility, lightweight, and wearability.

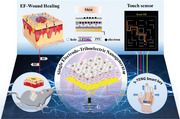

## 1 Introduction

The power of electricity contributes to every aspect of human innovation in an incredible way. In the recent era, portable miniaturized electronic gadgets provide easy access to global information and enable people to stay connected from almost anywhere. However, such portable electronic devices demand reliable and sustainable power sources. Plenty of green energy sources (wind, solar, mechanical vibration, and ocean waves) were utilized to fulfill this energy shortage. Among them, triboelectric energy nanogenerators (TENG) are prospective candidates for the future self‐powered energy source, which efficiently convert mechanical energy into electricity and provoke intensive research in various fields.^[^ ^1^, ^2^, ^3^ ^]^ TENG enable electricity by a coupled effect of tribo‐electrification and electrostatic induction effects. However, most of the high‐performance wearable TENG devices are constructed using rigid metal electrodes or conductive silicon‐based rubbers. Besides, such electrode films do not demonstrate air permeability, which may cause skin irritation due to sweating.

Wearable on‐skin TENGs are highly desirable along with good stretchability, air permeability, and waterproof capability.^[^ ^4^, ^5^ ^]^ Electrospun nanofibers and textiles‐based electrodes are ideal substrates to meet these requirements. It is also important to pay attention to their wearing comfort, environmental friendliness, and antibacterial ability.^[^ ^6^ ^]^ Despite the fact that electrospun NFs are lightweight and flexible, ultrathin nanofiber has a high surface area to volume ratio,^[^ ^7^ ^]^ and its irregular bumpy surface can generate more static charges than other normal films, making them still a competitive option in TENG fabrication. TENG devices are categorized into four modes namely, contact‐separation, single‐electrode, contact‐sliding, and freestanding.^[^ ^8^ ^]^ Among them, single electrode mode (S‐TENG) poses a thin device architecture with super agility and flexibility.^[^ ^9^, ^10^ ^]^ Moreover, fast response time with high voltage pulse replaces the traditional ceramic piezo sensors in motion detectors.^[^ ^11^ ^]^

Notably, the density of electrostatic induced charges in the friction layer has been focused on enhancing the electric output of TENG. Particularly choosing dielectric material with defects (porous, rough contact surface), incorporating the charge trapping materials, optimizing the gap between the friction layer and primary‐electrode and separating them with the charge trapping layer can enhance the surface charge density.^[^ ^12^, ^13^, ^14^ ^]^ For example, Kim et al. investigated the S‐TENG electrical output performance by varying the thickness of the PDMS deep charge trapping layer.^[^ ^15^ ^]^ Similarly, by applying a polyimide insulating thin layer as a separator the potential loss from the recombination can be prevented.^[^ ^16^ ^]^ These literature studies proved that reducing the charge recombination could directly enhance the surface charge and reach the saturation point rapidly. On the other hand, placing the high electron affinity materials as interlayer like reduced Graphene oxide,^[^ ^17^ ^]^ MXene,^[^ ^18^ ^]^ MnO nanosheets^[^ ^19^ ^]^ etc., act as a charge storage layer. However, direct contact with the electrode often leads to fast charge drifting due to the metal‐semiconductor Schottky contact. In light of these findings, the charge‐trapping mechanism could still be improved.

S‐TENG are suitable candidates for human‐machine interface energy harvesting and sensing applications, hence the animal skin and hair naturally generate electrostatic charge when approaching the opposite charged surface.^[^ ^20^, ^21^ ^]^ This phenomenal behavior can be utilized in many ways to produce free electric energy, and biophysical medical appliances with the help of electrical circuits. Recently, tissue‐engineering research achieved new milestones by improvising the trans‐epithelial potential (TEP) charges with exogenous EFs to treat chronic wounds.^[^ ^22^, ^23^, ^24^, ^25^ ^]^ TEP is the key component in wound repairing where the charged ions flux and cell transport effectively take place.^[^ ^26^, ^27^ ^]^ Although numerous conductive hydrogel energy transfer mediums have been used to transfer external electric current to treat chronic wounds, these patches rely on stable DC power battery resources.^[^ ^28^, ^29^ ^]^ The development of nanofiber‐based TENG is widely adapted by wearable smart garment fabrications and has become a true energy source for portable electronic gadgets.^[^ ^30^, ^31^ ^]^ Current developing research extensively investigates the optimization of EF strength and the unidirectional effect on wound remolding.^[^ ^32^, ^33^ ^]^ However, to further improve the visibility of TENG in wound management; it is paramount to specify the effect of tribo‐positive and negative potential charges with the TEP mechanism.

Herein, we propose a surface charge improved S‐TENG for biomechanical sensing and EF assisted wound healing applications. The TiO_2_‐MXene (TMx) incorporated PS NFM as a charge trapping layer benefited the electrical performance enhancement. Furthermore, the tribo‐negative layer PVDF/PU (PPU) NFM with improved mechanical properties promises a long‐lasting conversion of biomechanical energy into electricity. The fabricated S‐TENG was tested in powering LEDs, and charging a hygrothermograph. Moreover, the high sensitivity of S‐TENG facilitated the engineering of the Snakebite game with four S‐TENG as smart keys. Owing to the excellent flexibility, hydrophobicity, breathability, and lightweight, the wearable feature was established. This, flexible and wearable S‐TENG with no biological toxicity has promising wound healing applications. The EF simulation by S‐TENG was further verified by in vitro and in vivo studies. Thus, the multiple features and multi modal operation of this S‐TENG pave way in the field of energy harvesting as well as therapeutic research.

## 2 Result and Discussion

**Figure 1:**
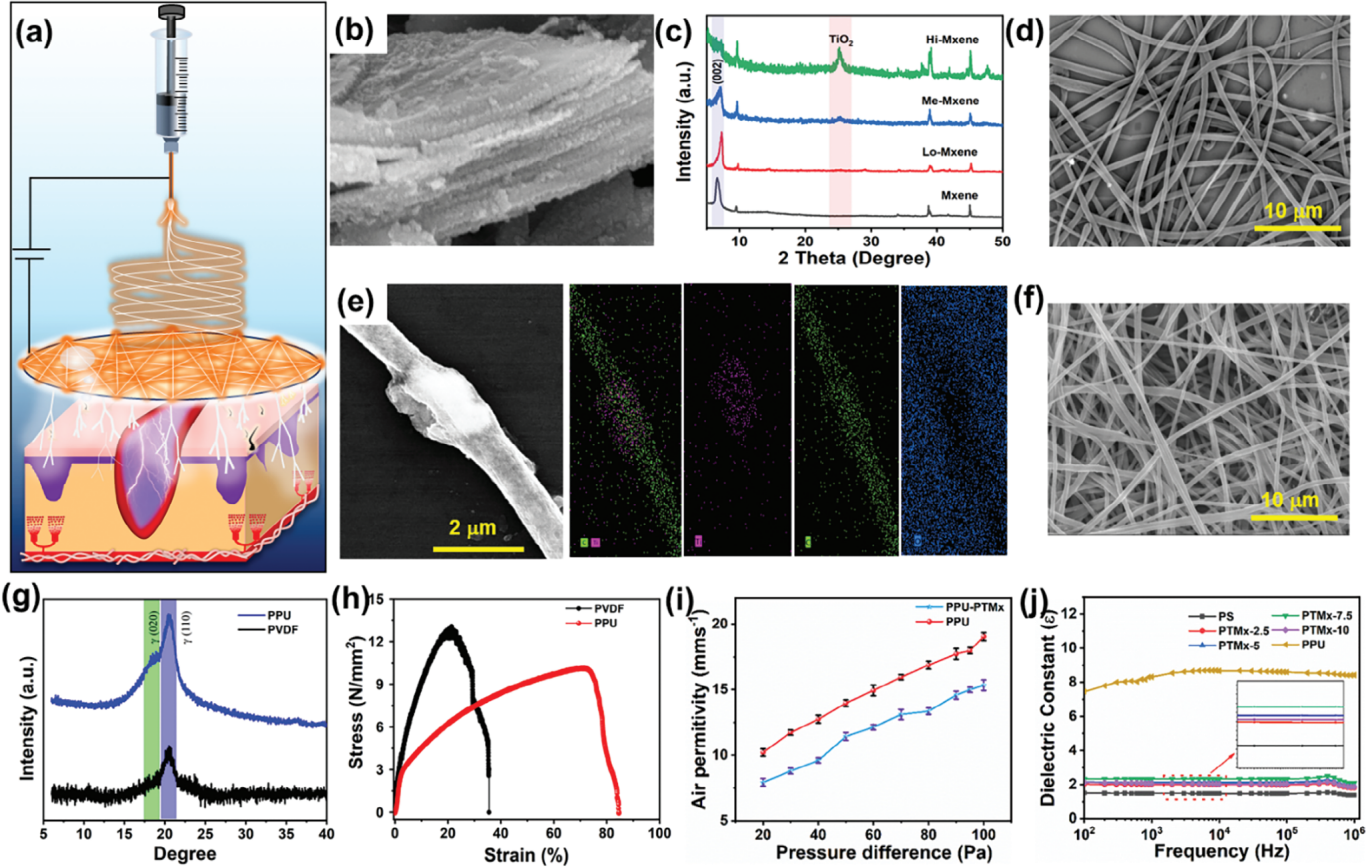
a) Schematically illustration of nanofiber synthesis and its EF induced wound healing application. b) FE‐SEM shows the surface morphology 12 h oxidized MXene. c) FT‐IR of TiO_2_ grown on MXene at 6, 12, 24 h. d) Surface morphology of PTMx nanofiber membrane. e) Magnified SEM image of PTMx with EDS elemental analysis. f) SEM image of PPU NFM friction layer (inset fiber diameter bar chart). g)) XRD spectra h) tensile strength of PVDF and PVDF/ PU. i) Air permittivity of NFM. j) Dielectric constant of the PPU, and PS with various doping amount of TMx.

**Figure** **1**a conceptually illustrates the electrospun nanofiber drawing and tribo‐electrostatic interaction of NFM onto the epidermis tissue for wound repair. As NFMs possess breathable, flexible and water permeability nature, they can efficiently convert human bio‐motion into electric energy without causing skin irritation. In prior, we have described the synthesis and structure morphology of TMx doping material using scanning electron microscopy (FE‐SEM) in Figure S1 (Supporting Information), the morphology images prove that oxidation was taking place on the MXene terminals. In particular, Figure 1b shows the partly oxidized MXene containing an optimal amount of TiO_2_ on the surface without destroying the entire carbon chain. X‐ray diffraction (XRD) in Figure 1c shows the peak positions corresponding to the MXene and TMx. More prominent peak appears at 6.9° indicating the successful etching of Al layers. It is evident that 6 h of oxidation does not show any of peak corresponding to TiO_2_ but in the case of 12 and 24 h oxidation, a new peak appears at 25° corresponding to anatase TiO_2_ (JCPDS 21‐1272) presenting partial conversion of MXene.^[^ ^34^ ^]^ From this, we conclude that 12 h oxidation renders a large surface area with an optimal amount of TiO_2_. Electrospinning process were carried out to prepare PS NF with different weight ratios of TMx. As shown in Figure S2 (Supporting Information) the pure PS polymer produces NFs with beads due to the lower viscosity, whereas TMx with optimal weight ratio (%) produces the beadles NFs. It is obvious that TMx doping has improved the localized electrical conductivity, which diminishes the beads efficiently. Despite this, at high concentration (10 wt%) of TMx exhibit needle block due to the particle agglomeration resulting in the less dense NFs formation. Figure 1d, FE‐SEM image with bar chart profile indicates the average diameter of 7.5 wt% of TMx doping (PTMx‐7.5) NFs to be approximately 800 nm and the uniform coverage of NFs. The magnified image confirms the MXene incorporation into PS NF, which was further confirmed by energy dispersive X‐ray analysis (EDS) mapping (Figure 1e). Also, the SEM surface morphology of PPU NFM given in Figure 1f and control sample PVDF in Figure S3 (Supporting Information) indicates that the diameter of the NFs was increased after blending PU because of viscosity change. The electroactive β phase of PVDF was improved via hydrogen bond formation with PU. As shown Fourier transform infrared spectroscopy (FTIR) (Figure S4, Supporting Information) spectrum at 762 and 840 cm^−1^ are attributed to the relative polymorphs of α and β phase of PVDF respectively.^[^ ^35^, ^36^ ^]^ XRD data (Figure 1g) result confirms the β crystalline phase appeared in PPU by suppressing the α‐phase.

Furthermore, a test sample with an area of 1.5×1.5 cm^2^ was subjected to stress‐strain measurements, as demonstrated in Video S1 (Supporting Information). The mechanical properties of NFMs are shown in Table S1 (Supporting Information); it is observed that the PU mixing increases the Young modules and elongation breaking point enormously (Figure 1h). Such enhanced properties can protect the NFM from delamination and mechanical damage during the pressure press‐release cycle, thereby expanding their potential lifespan. In addition, to meet the wearing comfort, the NFM scaffolds were subjected to air‐breathing tests, water‐vapor permeability tests, and water contact angle tests (WCA) individually. In Figure 1i, the air permeability of PPU NFM was shown without and with PTMx NFM interlayer; the obtained results indicated that both samples membrane has a linear relationship with air pressure (20–100 Pa). Similarly, water‐vapor permeability was evaluated by comparing the water vapor evaporation rate of NFMs with paper and cotton as shown in Figure S5a (Supporting Information). These results confirm the produced NFMs possess good air and water‐vapor permeation. Moreover, the WCA of PVDF NF increased due to the formation of hydrogen bonds by PU, which is in agreement with FTIR results. While WCA of PS NF was reduced owing to the hydrophilic nature of MXene, further the electrode styrene butadiene (SBS) NF WCA was measured, it is admired that all the WCA results show hydrophobic (≤90°) properties that sustain the NFM in sweat conditions.

In general, the electrostatic charge polarity of the TENG is defined by the high dielectric permittivity and low dielectric loss of the material. The generated EF would be strongly established at a high dielectric constant; for example, Dayananda et al.^[^ ^37^ ^]^ discovered that incorporating MXene into polymer builds the interfacial polarization and improves the dielectric constant of polymers. Taking this into account, the TMx was incorporated into PS polymer, and as expected the dielectric value of PS improved upon TMx concentration. Figure 1j, shows the dielectric of PS NFM substantially improved up to 7.5 wt% doping, which is near the percolation limit. However, the charge loss occurred when the TMx reached 10 wt% due to ohmic contact between the conductive fillers. It is obvious that the PPU NFM dielectric value is higher than the PS due to the PVDF naturally exhibiting strong charge polarity in β semi‐crystalline phase.^[^ ^38^ ^]^

### 2.1 Electrical Output Performance of S‐TENG

**Figure 2:**
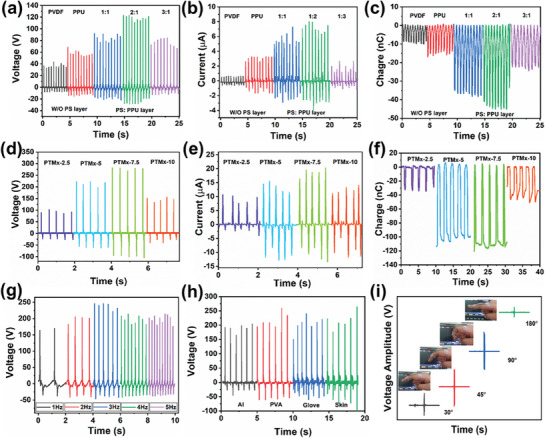
a) Optimization of voltage, b) short‐circuit current and c) charge transfer of S‐TENGs, pure PVDF and PPU friction layer; PS charge trapping layer with three different thickness (1:1, 2:1, 3:1). d–f) Influence of PS interlayer with various doping amount of TMx in electric output performances. g) Voltage of S‐TENG upon various applied frequency. h) S‐TENG working ability under various tribo‐positive friction materials. i) Finger mounted S‐TENG show voltage at different bending angles.

In **Figure** **2**, we have demonstrated the electric output performance of the TENG by optimizing the thickness of the charge trapping layer and doping amount of TMx. Previously, in the S‐TENG device fabrication and charge trapping mechanism illustrated in Figure S6 (Supporting Information), the addition of PU improved the tribo‐electronegativity of PVDF, resulting in an increment in the output performance (Figure 2a). Although, increasing the PVDF electroactive phase does not effectively improve the electric output of TENG, rather close contact with the primary‐electrode causes charge recombination and a weak EF. To prevent such energy loss and improve the static potential charge distribution, PS charge trapping polymer was placed between the contact material and the primary‐electrode. By fixing the 20 µm thickness of PPU NFM, we optimized the PS NFM thickness of 20, 40, and 60 µm (1:1, 2:1, 3:1 represented). As shown in Figure 2a–c), PS interlayer thickness has an influence on the results of open‐circuit voltage (*V*_OC_), short‐circuit current (*I*_SC_), and transfer charge (*Q*). According to Coulomb's law expression, the EF is inversely proportional to the square of separation distance (*r*) of the dielectric layer with an electrode. Therefore, at 20 µm separation distance, the induced charges are transferred and stored in the PS layer, which gives approximately *V*_OC_ = 80 V, *I*_SC_ = 5.5 µA, *Q* = 35 nC. However, at a high EF, the accumulated electrons rushed towards the electrode with high velocity. Subsequently, at 60 µm dielectric thickness provides a large surface to store the electrons, still the electrostatic potential is inversely proportional to distance; therefore it induces the weak surface potential charges on the electrode which gives *V*_OC_ = 60 V, *I*_SC_ = 2 µA, *Q* = 20 nC. While, 40 µm PS thickness exhibits the electrostatic charge with threshold limit giving *V*_OC_ = 120 V, *I*_SC_ = 8 µA, *Q* = 40 nC approximately. Enhancing the EF strength of the dielectric without losing charges would trigger the researchers to focus more on energy storing semiconductors addition. MXene has already proved its potential in energy storage and optoelectrical applications, due to its large surface area and high electrical conductivity. In contrast, in situ oxidation provides the MXene with improved dielectric properties, which helps to store charge without loss. However, at high EF the stored charges flow to the nearby electrode, whereas adding this charge carrier into the PS dielectric polymer can accumulate the charges at the interface owing to the Maxwell‐Wagner Sillars interfacial polarization phenomena. As proof of concept, Figure 2d–f shows the electric output performance of S‐TENG, when the TMx doping ratio of 2.5, 5, and 7.5 wt% to PS, the output performance increased gradually. The maximum output [*V*_OC_ = 280 V, *I*_SC_ = 20 µA, *Q* = 120 nC] was achieved with a doping amount of 7.5 wt%. At this level considered the percolation limit, where the induced charges transfer to TMx and are trapped. This result confirms that TMx doping retains the electron clouds even at the high EF, which forms the maximum static charges on the primary electrode. On the contrary, further addition of TMx (10 wt%) was not helpful because high doping of conductive material forms an interconnecting network that leads to electron flow through them. Moreover, the particle agglomeration causes poor fiber morphology with less dense NFM per unit area, it exhibits a decreasing trend in electrical outputs. A frequency range of 1 to 5 Hz was applied to study the change in V_OC_ with changing frequency as shown in Figure 2g. Notably, the maximum voltage output was obtained at 3 Hz frequency. The reason is that at higher frequencies, the contact‐separation operation time becomes shorter, which results in a faster surface charge saturation. In spite of this, further increasing the frequency does not help to deform the S‐TENG efficiently and induces charges, so the *V*_OC_ starts to decrease. The contact electrification ability of PPU NFM with various positive friction layers such as Al, PVA, glove and skin was also investigated (Figure 2h). The variation in the V_OC_ is related to their electron donating nature, a high number of electrons causes strong polarization, which gives high amplitude output. Moreover, a prototype of S‐TENG device with 1 × 3 cm^2^ was mounted on finger to study the sensitivity at various bending angles (30°, 45°, 60°, and 90°) as shown in Figure 2i. It was observed that the results were in linear agreement with strain based upon the bending angle.

### 2.2 Surface Charge Enhancement in S‐TENG

The surface charge enhancement in TENG can be directly related to the built‐in EF of the dielectric. In detail, the contact separation or sliding mechanism of TENG generated the electrostatic potential difference between the electrode and contact layer where the strong EF is built. However, the induced EF could vanish rapidly, which affects the surface charges on the contact layer. This phenomenon can be explained by mainly two factors, i) neutralization of surface charges by ionized air, ii) at high EF the charges drive faster to the electrode. Owing to the above reasons, the maximum charge transfer efficiency of S‐TENG is less than the normal TENG. Many physical and chemical surface treatments were performed to improve the tribo‐electrified charges on the electrified area.^[^ ^39^ ^]^ However, to achieve high performance S‐TENG the induced electrons must be trapped and stored within the dielectric medium. As we discussed previously, the PTMx interlayer increases the capacitance of the transfer charge accumulation by metal‐polymer interfacial polarization and prevents the charge drifting by the electrode, which enhances the surface potential (*Q*) and surface charge density (*σ*) on the electrode. According to the equivalent circuit diagram, the voltage output (*V*) is a linear relationship with *Q* and reciprocal to the capacitance (*C*),^[^ ^40^, ^41^ ^]^

(1) $$V=-\frac{1}{C}\times Q+V_{\mathrm{oc}}$$

where *V* is the sum of the *E* = *E*_PPU_ + *E*_PTMx_ of the dielectric layers, in short circuit condition *V* equals 0 and *Q* equals to short circuit transfer charge (*Q*sc), therefore,

(2) $$Q\mathrm{sc}=CV_{\mathrm{oc}}$$

Due to the fixed electrodes in S‐TENG, the *C* value becomes constant even with the increase of gap distance *x*(*t*), resulting in the increase of surface charge density (σ) on the electrode leading to a faster saturation of *V*_OC_.

(3) $$V_{\mathrm{oc}}=\frac{\sigma x\left(t\right)}{\varepsilon_{0}}$$

**Figure 3:**
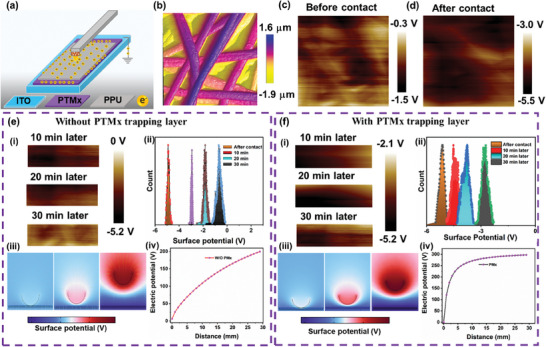
a) Schematic representation of KPFM measurement on PPU surface with and without charge trapping PTMx‐7.5 NFM layer. b) Topographical image of PPU frictional layer. c,d) Surface charge density on PPU layer before and after contact separation process. e‐i) shows the observed surface charge loss on the friction layer without PTMx‐7.5 NFM. (ii) The surface potential derived from the image (i). (iii‐iv) COMSOL derived theoretical assumption on electric potential. f‐i,ii) surface energy loss, (iii,iv) COMSOL derived theoretical electric potential with PTMx‐7.5 NFM.

To understand the proposed theoretical model, evaluating changing *Q* on the dielectric surface as a function of time is a more promising approach. In **Figure** **3**, we have demonstrated the comparison of overall surface charge distribution on PPU NFM with and without PTMx trapping interlayer over time by utilizing a powerful Kelvin probe force microscope (KPFM) analytical tool and COMSOL multiphysics simulation study. As systematically described in Figure 3a, a KPFM superior lateral resolution technique was employed to capture the surface image and quantify the *Q* variations at the nanometer scale. As following our previous study,^[^ ^41^ ^]^ the potential charges were induced by gently rubbing the surface of contact material with the finger, resulting in the generation of high surface charge density on the NFM. Figure 3b–d shows the PPU NFM surface topographical image with respective charge potential distribution before and after contact separation with skin. The obtained spatial potential value of (−1.5 V) was decreased to (−5.5 V) after the surface contact experiment. A negative sign in the obtained value confirms the polarized charges generated on the PPU NFM resulting in the enhancement in tribo‐negativity, thus generating the opposite charges on the electrode surface. As a result, a strong EF forms between the electrode and the dielectric. However, inevitable charge recombination at the electrode surface neutralized the total charge transfer efficiency, which could reduce the S‐TENG performance. To address the potential loss, screening the generated electrons inside the dielectric can significantly change the electrical output. By considering this, we trapped the charges into the PTMx NFM and avoided the charge recombination with the electrode.

Usually KPFM measurements, the surface potential is derived from the contact potential difference between the AFM measuring tip and the sample surface, which defines the work function of the sample.

(4) $$V_{\mathrm{CPD}}=\left[\varphi_{\mathrm{sample}}-\varphi_{\mathrm{Tip}}\right]\;/e$$

where φ_sample_, φ_Tip_ are the work function of the sample and the tip, *e* is the elementary charge.

The above equation confirms that the obtained result during the operation (after contact with skin) also corresponds to the PPU sample work function. By taking this into account, we measured the retained charges on the surface before and after contact. As well the surface charge dissipation was tracked at various time intervals of 10, 20, and 30 min in a closed environment to avoid the influence of moisture ionization (Figure S7, Supporting Information). Therefore, the potential loss in contact surface area can only be caused by charge neutralization with the electrode. In Figure 3e without PTMx interlayer, the surface electrons disappear faster due to the above‐mentioned factor. Figure 3e‐i–iii, experimental results show that after 30 min interval the surface potential value has increased from −5.1 to −1.5 eV. This confirms that the surface electron loss is because of charge drifting resulting in poor polarity. Whereas, after placing the trapping layer the induced electrons are trapped within the PTMx layer, which prevents and slower the rate of charge recombination. Meanwhile, the stored electrons create a strong EF to form high charge polarization (Figure 3f).

A simulation study was performed using COMSOL (6.1) to conclude the experimental results supported with theoretical proof. Herein, the S‐TENG model is defined with and without PTMx trapping layer, the PPU contact layer with human skin. The derived surface potential values of Figure 3e‐iv,f‐iv proved that inserting the trapping layer would increase the S‐TENG performance. In summary, we conclude that insertion of the PTMx charge trapping layer not only prevents energy loss but also increases the EF strength, which induces high polarized charges on the primary electrode. This new approach can give the solution for fabricating high‐performance TENGs without energy loss.

### 2.3 Evaluating the Electrical Performance of S‐TENG in Commercial Electric Appliances

**Figure 4:**
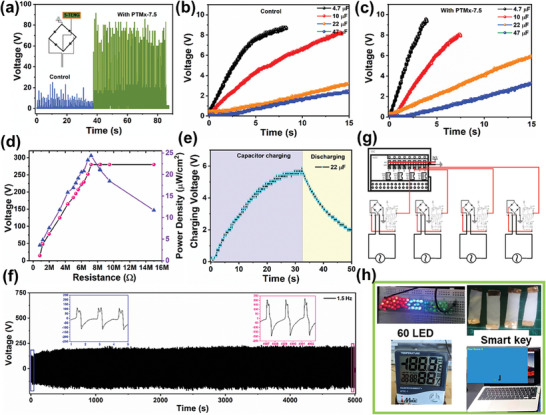
a) Rectified voltages. Inset circuit shows the bridge rectifier with S‐TENG device. Capacitor charging ability of S‐TENG b) without and c) with PTMx‐7.5 NFM interlayer. d) Power density of the optimized sample under various resistance. e) Powering the time piece using 22 µF capacitor charging. f) S‐TENG duration test under 1.5 Hz for long time, inset (before and after voltage output). g) Engineering of electronic circuit for self‐powered flexible smart key. h) Photographical images of powered electronic gadgets, smart keys and “Snakebite” video game.

**Figure** **4** illustrates the utilization of electrical output performance in electrical appliances and real‐time demonstration of S‐TENG in touch sensors. In general, the contact separation process of TENG device produces the electrostatic potential charges in terms of alternating current (AC). The AC produces a high amplitude voltage, due to the fact that the parallel plate capacitors are charged and discharged simultaneously. In order to rectify this, a four‐wave bridge rectifier was connected in the series, as described in Figure 4a inset circuit sketch. The results confirm the full conversion of AC sine‐wave signal into direct current (DC). To demonstrate the working of S‐TENG in portable electronics, our device is connected to charge various capacitors (4.7, 10, 22, 47 µF) by applying the pressure at a constant (3 Hz) frequency. Figure 4b,c render the comparison of capacitor charging efficiency of S‐TENG without and with PTMx NFM interlayer, respectively. It is obvious that after inserting PTMx NFM the charging time becomes shorter. From the above results, we concluded that with the trapping layer, the surface charges attain their maximum shortly and facilitate sustainable output for rectification with applied frequency. Before employing the S‐TENG directly to charge electrical appliances, we evaluated the sustainable power density (PD) across the various load resistance (*R*) (1–100 MΩ). As Ohm's law states, with an increasing *R*‐value the prompt voltage increases, when *R* is significantly high the *V*_OC_ becomes saturated. In such manner, the maximum PD reached to be 25.2 µW cm^−2^ at 7 MΩ resistance, and further increase of *R* leads to the PD decline as shown in Figure 4d. To demonstrate the S‐TENG performance towards electronic gadgets, we have shown the instantons powering of 60 LED (red‐green‐blue) in Video S2 (Supporting Information) this proves that the device produces enough power with regular periodic pressure. Similarly, an experiment with a capacitor was conducted by connecting the S‐TENG to a circuit containing a digital hygrothermograph device (Figure 4e), this graph reveals a typical charge‐discharge can occur in repeated cycles. Furthermore, the device durability was tested by continuously beating with a homemade tapping instrument at a fixed 1.5 Hz frequency. As displayed in Figure 4f, the voltage profile appeared regularly, confirming that S‐TENG can generate stable output for a long‐term. As shown in the photographic image in Figure S8 (Supporting Information), after long‐time tapping the PVDF NFM displays a poor surface with delaminated fibers, whereas the PPU NFM surface remains intact. This outcome proved that the PPU NFM can protect the PTMx interlayer and is extensively operative when employed for human use.

Featuring a high electronegative surface, our S‐TENG exhibits excellent energy harvesting properties on human skin and epoxy gloves. In order to make S‐TENG devices in biocompatible wearable applications, the device performance with various area dimensions was evaluated. According to Figure S9a (Supporting Information) with increasing the friction area the energy output increases, which is obvious that high surface area can accumulate a large charge density. Additionally, S‐TENG device shows excellent sensitivity under various load impact pressure (Figure S9b,c, Supporting Information). Herein, we applied force of 0.5 to 15 N m^−1^ with load mass, the observed voltage amplitude shows an increasing trend. From the obtained result, the derived sensitivity value of S‐TENG is 11.2 V N^−1^. Furthermore, the device responds to the other regular environmental forces of finger‐taping, metal friction and water droplet impact pressure (Figure S9e–g, Supporting Information). Owing to S‐TENG high sensitivity, we designed self‐powered physical sensors using circuit‐engineering technology. As shown in Figure 4g, four S‐TENG devices were constructed as flexible keys to the programmed Snakebite video game (Video S3, Supporting Information). The devices with high sensitivity perform well as self‐powered navigation keys. This proto‐type self‐powered invention can reduce battery usage in regular switches and gaming gadgets (Figure 4h). Moreover, AgNP‐coated SBS NFM (Ag/SBS NFM) electrodes with stretchable, breathable and antibacterial properties afford S‐TENG to be utilized for skin wearable devices.

### 2.4 Influence of S‐TENG Electric Field Simulation on L929 Cells Vitro Experiment

**Figure 5:**
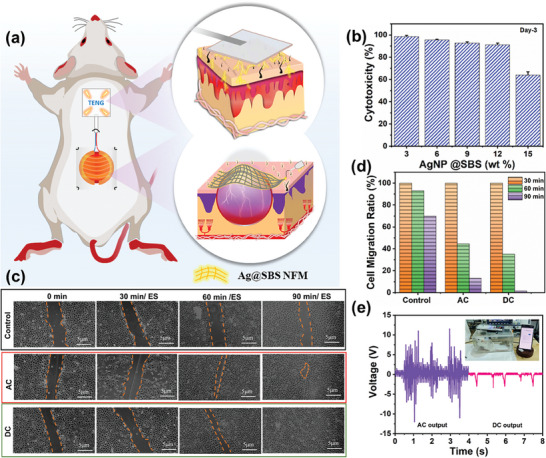
a) Illustration of S‐TENG electric simulation during wound healing process. b) Cell viability of Ag/SBS NF with different doping amount. c) In vitro starch assay of L929 cells without treatment (control) and with AC/DC EF. d) Cell migration ratio before and after 30, 60, 90 min. e) S‐TENG electric outputs with open circuit and rectified voltage.

Prior to EF stimulated wound healing experiment, it is important to understand the basic healing mechanism of wounds. Normally wound healing process is promoted by difference in TEP between the wounded area and normal skin.^[^ ^42^ ^]^ Comprehensively, when a wound occurs, the endogenous TEP generates an electric current to drive the charged ions Na^+^ Cl^−^ toward the center of the wounded area to promote the healing process.^[^ ^22^, ^27^ ^]^ However, in treating chronic wounds, a weak TEP prolongs the wound healing duration. This creates the demand for the use of an exogenous EF. As shown in **Figure** **5**a, the S‐TENG was designed with a PET substrate for free tapping on the rat skin when it moves (Figure S10, Supporting Information), and the induced energy was transferred to the wounded area with the help of Ag/SBS NFM. A breathable Ag/SBS NFM was fixed on the wound using transparent 3M Tegaderm. Our previous work on Ag/SBS NFM electrode synthesis with optimized electrical performance and antibacterial properties was followed up in this work.^[^ ^41^, ^43^ ^]^ In addition, we directly investigated the cytotoxicity of the electrode membrane with L929 cells by adjusting the AgNP coating percentage of 3, 6, 9, 12, and 15 wt% (Figure 5b). The obtained results show that up to 12 wt% of AgNP, the electrode shows good agreement with 90% cell viability. However, further increasing the AgNP concentration is not advisable since it shows electroporation in the nucleus. Using the L929 cell assay, an vitro scratch experiment was conducted to examine the influence of the EF on cell migration and assembly. As we discussed in the experimental section a linear scratch was made on the cell monolayer with a spacing of approximately 5 µm. Afterwards, a pair of copper taps were fixed on both sides of the scratch with a distance of 1.5 cm, and a continuous AC EF was given by the TENG device. The sewing machine was used to tap the device with 4 Hz frequency and continue for 30 min, this procedure was continued for three consecutive days. A similar procedure was followed for the DC EF; the experimental procedure was captured in Video S4 (Supporting Information). A control sample was kept aside without any special treatment. As depicted in Figure 5c, the microscopic result ascertained that EF stimulation has improved cell migration enormously. After 30 min of EF simulation, the AC potential accelerate the cell mobility randomly which was clearly observed, whereas the DC drove the cells uniformly to the scratch. Meanwhile, the control cell group remained unchanged. Typically, the EF strength in the units of V m^−1^ may cause electroporation in vitro experiments, but in the living body, it is safe and harmless to the cells. However, this time‐limited operation accelerated the cell regeneration faster without causing the electroporation in the nucleus. Figure 5d, given the quantitative assessment of the cell migration ratio of the three test samples, in which DC EF results show 100% cell rearrangement; AC EF shows more than 90% regeneration. Before employing the device in vivo experiment, we evaluated the AC and DC electric output performance of the skin‐mounted S‐TENG. In Figure 5e, the obtained result elicits that frequent voltage pulse was obtained when the rat was physically active (running, bending, shaking), and the obtained *V*_OC_ is 1‐10 V and rectified negative bias voltage is 1–2.5 V. To support this, we have monitored the rat movement by using Arduino Uno wireless circuit, Video S5 (Supporting Information) indicates the “Wound Healing Progress” keyword to represent the S‐TENG activation. It is assumed that our S‐TENG's EF stimulation cues could assist wound re‐epithelialization rapidly.

### 2.5 Electric Field Accelerated Wounded Repairing

**Figure 6:**
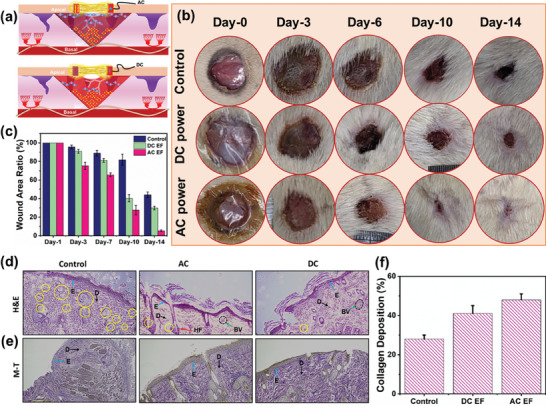
a) Schematic demonstration of AC and DC ES process on wounded area. b) Photograph of wounded skin at various time intervals. c) Wound area recovery after 3, 7, 10, 14 d of treatment. d) H&E and e) M–T histogram examination of newly formed skin tissue. f) Collagen density measurement of M–T image.

Our S‐TENG device is believed to induce triboelectric charges continuously when in contact with the rat skin, as formed electrostatic potential progressively provokes cells to proliferate towards re‐epithelialization. To prove this, an vivo experiment was conducted by directly mounting the S‐TENG on the wounded rats as described in the schematic **Figure** **6**a. According to the animal experiment protocol (LAC‐2022‐0203), six SD rats' dorsum skin was cut into 1.8 cm in circles. A pair of rats were used for the concordant results. The wound status was monitored by taking photos on 3, 7, 10, and 14 d of surgery (Figure 6b). The control sample represented rats without being subjected to any treatment.

Generally, the wounded skin naturally produces TEP to pump the Na^+^ ions inwards and Cl^−^ ions apically outwards, where the short‐circuited positive current flows towards the wound center. Lately, it has been proven that increasing ion flux in endogenous EF by applying exogenous current works well. The purpose of the present study is to investigate how changing exogenous electric potentials affect the flow of endogenous ions movement. During AC EF simulation, the induced electrostatic potential generates dense positive surface charges on the electrode whereby high Cl^−^ ions were generated at the apical side of the wounded area as shown in Figure 6a. As generated exogenous potential, induce the large endogenous EF as favoring the Na^+^ ion at the basal side (unwounded). It is evident that when coupling an exogenous EF to TEP accelerates wound healing rapidly, besides forming laterally orientated cell proliferation. We patch the electrode mainly to contact the edges of the wound this allows the wound to recover symmetrically. The obtained wound closure ratio already reached 95% on day 14 (Figure 6c). This result ascertains that tissue granulation occurred from all directions (Figure 6b). Whereas in DC EF simulation, the transformed DC negative bias via bridge rectifier was transferred to the electrode patch (Figure 6a). As we expected the negative potential significantly affects the TEP. This may be the case that Cl^−^ ions do not attract the negative charges, thus generating a poor potential barrier at the skin surface. However, when compared with an untreated wound, DC EF simulation significantly improved wound healing by 70%.

After the completion of 14 d of the experiment, the regenerated skin was collected from the rats for histological analysis in order to quantify the phase of proliferation, tissue granulation, and collagen formation. Hematoxylin and eosin (H&E) staining experiment results in Figure 6d reveal the EF simulated the samples have progressively improved cell proliferation including the formation of thick keratinocytes (dark purple) at the epidermis layer and blood vessels (BV‐black circle) at the dermis (D) region. Especially with some new hair follicles found in the EF treated wounded area, this confirms the complete rehabilitation of the epidemic skin. On the contrary, the control sample shows many gaps, and a large number of inflammatory cells in the dermis, this indicates that granulation was not taking place in the control sample. Masson trichrome (M‐T) staining test results as seen in Figure 6e further affirmed the dense collagen formation upon EF simulation, whereas the control sample shows defects, which affect the elasticity of the skin. Using imageJ software RGB color deconvolution to measure the collagen deposition of M‐T samples. As shown in Figure 6f, collagen deposition of approximately 28%, 41%, and 48% in control, DC, and AC test samples respectively. These results confirm that electrostatic potential significantly promoted fibroblast cells for collagen formation to accelerate wound healing.

## 3 Conclusion

In summary, we improved the S‐TENG device surface charge density and output performance through a new regulated charge‐trapping mechanism of interfacial polarization. Through the intercepting of PTMx NFM, we were able to increase the charge‐storing capacity as well as the resistance to charge recombination. Further, all fiber‐based fabrication offers many advantages for wearable biomechanical energy harvesting. The tribo‐friction layer PPU shows good mechanical properties and stretchability which extend the duration of S‐TENG lifespan. With excellent flexibility, hydrophobicity, breathability, and lightweight, this TENG is highly comfortable to wear. The electrical output performance of S‐TENG was evaluated in glowing 60 LED lights, and commercial capacitors. Intriguingly, owing to its high sensitivity the S‐TENG was designed as smart keys for “Snakebite” video game. In addition, the resulting output was utilized to treat chronic wounds, where the EF was induced through connecting. Ag /SBS NFM. Prior to this wound healing experiment, the efficiency of S‐TENG was verified by in vitro and in vivo tests. The device tested in both AC and DC modes of operation showed better results. Thus, the multiple features of S‐TENG with wearable advantage are expected to have a significant impact in sensors as well as therapeutic medicinal areas of research.

## Conflict of Interest

The authors declare no conflict of interest.

## Supporting information

Supporting Information

Supplemental Video 1

Supplemental Video 2

Supplemental Video 3

Supplemental Video 4

Supplemental Video 5

## Data Availability

The data that support the findings of this study are available from the corresponding author upon reasonable request.
