# Supplementary material for: Rationally Improved Surface Charge Density of Triboelectric Nanogenerator with TiO2‐MXene/Polystyrene Nanofiber Charge Trapping Layer for Biomechanical Sensing and Wound Healing Application
Source: Adv Sci (Weinh). 2024 Jul 9;11(34):2404019. doi: 10.1002/advs.202404019 (PMC11425989; doi:10.1002/advs.202404019)
Supplement: Supplementary file 1 — Supporting Information [file ADVS-11-2404019-s002.docx]

**Supporting information**

**Rationally Improved Surface Charge Density of Triboelectric Nanogenerator with TiO_2_-MXene/Polystyrene Nanofiber Charge Trapping Layer for Biomechanical Sensing and Wound Healing Application**

Manikandan Venkatesan ^a^, Jayashree Chandrasekar ^a^, Yung-Chi Hsu ^a^, Ting-Wang Sun ^a^, Po-Yu Li ^a^, Xuan-Ting King ^a^, Ming-An Chung ^b^, Ren-Jei Chung ^c^, Wen-Ya Lee ^c^, Ye Zhou ^d,*^, Ja-Hon Lin ^e,＊^, and Chi-Ching Kuo ^a,f,*^

^a^ Institute of Organic and Polymeric Materials, National Taipei University of Technology, Taipei 10608, Taiwan.

^b^ Department of Electronic Engineering, National Taipei University of Technology, Taipei 10608, Taiwan.

^c^ Department of Chemical Engineering and Biotechnology, National Taipei University of Technology, Taipei 10608, Taiwan.

^d^ Institute for Advanced Study, Shenzhen University, Shenzhen 518060, P. R. China.

^e^ Department of Electro-Optical Engineering, National Taipei University of Technology, Taipei 106, Taiwan.

^f^ Advanced Research Center for Green Materials Science and Technology, National Taiwan University, Taipei 10617, Taiwan

^*^Author to whom all correspondence should be addressed

Tel: 886-2-27712171*2407 Fax: 886-2-27317174

Correspondence Author: Prof. C.-C. Kuo (E-mail: [kuocc@mail.ntut.edu.tw](mailto:kuocc@mail.ntut.edu.tw))

Prof. J.-H. Lin (E-mail: [jhlin@ntut.edu.tw](mailto:jhlin@ntut.edu.tw))

Prof. Y. Zhou (E-mail: [yezhou@szu.edu.cn](mailto:yezhou@szu.edu.cn))

**Materials and Methods**

**Materials and Instrumentation:**

MAX phase MXene and LiF were purchased from Sigma-Aldrich. Polyvinylidene fluoride (PVDF) pellets (275,000 g/mol), Polystyrene (PS) (280000 g/ mol), Styrene butadiene (SBS), Ag nanoparticle precursor, and Hydrazine were purchased from Sigma-Aldrich. Polyurethane (PU) elastollan 1185A 10w000 was purchased from BASF, Taiwan. All the materials were utilized directly without any further purification. Solvents N, N-dimethylformamide (DMF), tetrahydrofuran (THF), ethanol, and hydrochloric acid (HCl) solvents were purchased from Sigma-Aldrich. Deionized water (DI) water.

Fourier transform infrared spectroscopy (FTIR) was performed on a spectrum Two (PerkinElmer, UK) in the region of 4000–400 cm^-1^ with 16 scans at a resolution of 4 cm^-1^. X-ray diffraction (XRD, PANalytical diffractometer (X’ Pert3 Powder)) techniques were used to study the XRD patterns of polymer composites. A tensile test QC 508 (Cometech Testing Machines Co., Ltd., Taiwan) was conducted to determine the mechanical properties. The water contact angle was measured using an FTA125 contact angle analysis system (FTA). Air permeability (TEXTEST FX 3300). Surface morphology of composites were studied by using scanning electron microscopy (SEM, JEOL JSM-6700F). Surface potential was observed using Kelvin probe force microscope (KPFM Bruker-Dimension Icon) and electrostatic voltmeter. The capacitance was collected using an LCR meter (Agilent E498E precision). Electrical performances such as the open-circuit voltage and short-circuit current were studied through Keithley (2634B) and Oscilloscope.

**Synthesis of In-situ Modified MXene:**

The MXene was obtained by selective etching of Al layers from MAX phase MXene powder using HCl liquid phase delamination technique. Typically, 1g of LiF powder was added to 10 ml of 6 M HCl and continuously stirred for 5 min to form uniform dispersion. Subsequently, 1.6g of MAX phase was carefully transferred into the solution and the reaction temperature was maintained at 35 ͦ C for 24h. After the etching process, the solution was centrifuged with DI water until the neutral pH value was attained. The as-obtained multilayered MXene was delaminated by ultrasonication for about 1 hour. Then, the resultant was again centrifuged and dried in a vacuum oven overnight. Finally, the dark powder obtained was filled with N_2_ gas and stored in a refrigerator for future modification.

The oxidized MXene was prepared by following the procedure,^[1]^ Followed by TiO_2_- MXene (TMx) obtained in situ oxidizing taking place under different time intervals. As derived MXene (3 wt %) disperse into 50 ml of water and kept stirring for 6h (Low oxidized), 12h (Medium oxidized) and 24h (high oxidized) respectively.

**Preparation of TiO_2_-** **MXene doped PS Nanofiber:**

A different weight ratio of (2.5, 5, 7.5, and 10 wt%) of 12h oxidized MXene powder was dispersed in DMF(99% purity) solvent and subjected to probe sonication for 0.5h to obtain the uniform dispersion. Then the PS pellet (10 wt%) was mixed with the above solutions by placing on the 60 ͦ C hot stirrer plate for 2h. The as formed, solution was filled into the electrospun syringe and kept at 15 cm distance from the nanofiber collector plate. The voltage regulator was adjusted to 18kV and the applied force was optimized to 0.5 mL/h. Finally, the electrospun process was carried out under 35% humidity and RT conditions. For simplicity, the resulting NFM products were named according to the MXene wt% as PTMx-2.5, PTMx-5, PTMx-7.5 and PTMx-10.

**Preparation of PVDF/PU Nanofiber:**

Tribo-negative friction layer PVDF/PU (PPU) NFM was prepared by mixing (PVDF: PU) in 3:1 ratio based on the literature survey. 15 wt% of PVDF solution was prepared by adding PVDF pellets into DMF/THF binary solvent and stirred for 2h at 60 ͦ C. Then, 8 wt% PU solution was obtained by adding PU pellets to the DMF solution and stirred for 4h at 60 ͦ C. Finally, the composite solution was electrospun with fixed parameters applied voltage, distance from the needle to collector and flow rate as 13 kV, 15 cm, 0.8 mL/h respectively. Similarly, the control sample (PVDF) NF was produced without the addition of PU.

**Fabrication of S-TENG Device with Flexible Electrode:**

To fabricate the flexible S-TENG device, the charge trapping PTMx NFM was collected directly on a pre-prepared AgNP-coated SBS stretchable electrode (primary-electrode). Consequently, the electronegative friction layer PPU NFM was collected on the PTMx layer. A Copper tape was used to extend the electrode in order to connect with the instrument. The reference electrode was connected to the ground. To acquire the electrical outputs various tribo-positive materials such as Al, PVA, epoxy gloves, and human skin were used.

**Nanofiber Membrane Breathability Test:**

To fabricate all fiber-based TENG for biocompatible wearable applications; it is necessary to evaluate the water vapor permeation. To perform this study, 5g of water was taken in 5 different test tubes, which were completely covered by (PPU-PTMx, SBS) NFM and cotton, paper was used for comparison, and then all the samples were kept under 35℃ under (35-40%) humidity for 3 days. The weight variation in the water was recorded every 6h, similarly, the control (without covering) was also recorded. The obtained results show the S-TENG NFM have good water vapor permeability.

**COMSOL Simulation:**

Herein, a theoretical evaluation of surface potential charge upon contact separation mode is demonstrated. The sample geometry parameters were fixed based on the original size area and thickness of 50 mm × 50 mm, and 0.5 mm respectively. The material geometry top-down order of skin (positive layer/ PPU (negative layer)/ PTMx (Trapping layer), Primary electrode, reference electrode). The top contact surface of PPU and bottom surface of the primary electrode are given as floating potential, and the reference electrode is placed right bottom of the primary-electrode with some gap in which the bottom surface is given as ground. The physics control mesh used extra fine meshing.

**Biocompatibility Evaluation (CCK-8 assay):**

The cytotoxicity of conductive electrode AgNW-coated SBS NFM on L929 mouse fibroblasts ( ATCC, USA) was evaluated by means of 3-(4,5-dimethylthiazol-2-yl)-2,5-diphenyl tetrazolium bromide (MTT) assay. To perform the cell viability, 4, 6, 8, 10, 12, and 14 wt% of Ag-coated SBS NFM samples were initially sterilized by dispersing them into 70% ethanol and exposing them to UV light for two hours, followed by addition of PBS to completely remove the ethanol. To prepare the medium for cell culture, the specimens were added to Dulbecco’s Modified Eagle’s Medium (DMEM) with 10% PBS (specimen: DMEM = 1:9). After cell-cultured for 24h, the cells reached confluency, then medium was centrifuged. The extracted cell trypsinized was dispersed into a 2 mL culture medium, and then the cells were counted using a hemocytometer. The counting resulted in about 1x10^4^ cells per well-seeded scaffold in 24h well plates which were placed in the incubator for cell growth on the fibrous scaffolds one day prior to the experiment ^[2]^. Consequently, the old medium was removed and DMEM containing specimen extract was added to the cells on the day of the experiment. Then, the cell viability was studied using MTT assay through the metabolic activity of the cells after a duration of 24h. The cell viability is calculated according to the OD value. The calculation formula is as follows:

Cell viability =$\frac{{OD}_{sample}-{OD}_{blank}}{{OD}_{control}-{OD}_{blank}}*100\%$

Where OD sample is the absorbance value of the sample group; OD blank is the absorbance value of the blank group; OD control is the absorbance value of the control group.

**Assessment of Cell Migration under AC/DC Electric Simulation:**

An in-vitro-scratch assay study was performed under the EF simulation. L929 cells at a density of 5 x 10^4^ cells were previously seeded in µ-Dish 35 mm well and culture for 24h at 37 ℃. An in-vitro scratch was created at the center of a well dish using a 500 μm thick doctor blade. Two copper electrodes with an area of 1x1 cm^2^ were placed on both sides with a distance of 1.5 cm so that cell migration is faster in the electric field. S-TENG output (AC/DC) energy passed through the electrode pairs continuously for 30 min, this experiment was conducted for three consecutive days. A control sample (without EF simulation) was also performed in a similar manner. Cell migration was monitored using an optical microscope. ImageJ software was used to calculate the area of the scratch of each optical image.

$$Cell migration ratio A\left( \% \right)=\frac{A_{0}-A_{s}}{A_{0}}\times100$$

Where ($A_{0}$) is the initial scratched area and $A_{s}$ is the healing scratch.

**In Vivo Test:**

As part of the EF simulation wound healing experiment, six Sprague Dawley (SD) rats (6 weeks of age) were used and a proper diet was fed. The experiments were carried out by following the guidelines of Institutional Animal Care-approved experimental protocols (LAC-2022-0203) and the Use Committee of Taipei Medical University. SD rats were anaesthetized by administering 0.3 ml of Zoletil 50:Rompun 20 drug solution with a ratio of (1:2) via intraperitoneal, then shaved dorsum skin was cleaned with alcohol and carefully removed with an area of 18 mm. Immediately, UV sterilized breathable, conductive Ag/SBS NFM electrode covered the wound area and fixed it with 3M™ Tegaderm™, then the electrode patch was charged via S-TENG (Fig. S). The tribo-electrified charges were generated when the S-TENG friction layer repeatedly contacted the hair/skin of the rat. Here, the physical movement of the rat was converted into electric output to simulate the wound healing. A digital oscilloscope was used to monitor the electrical pulse. Six wounded SD were divided into three groups, and received the following treatments: group 1, control (without treatment); group 2, applied AC potential; the group 3, DC rectified voltage. Wound healing area was observed at different time intervals of day 0, day 3, day 6, day 10, and day 14 using digital photographs. To quantify the area of wound healing imageJ software was used. At each observation time point, the Ag/SBS electrode was replaced with new membrane.

$$Wound healing (\%)=\frac{Wound area \left( A_{s} \right)-Initial wound area \left( A_{0} \right)}{Initial wound area \left( A_{0} \right)}\times100$$

After completing the 14 days of the experiment, rats were euthanized using CO_2_ gas and the wound area was excised down to the fascia for histological examination. The cut skin samples were preserved in 10% formalin and stored by standard paraffin embedding procedure. Hematoxylin and eosin (H&E) and Masson’s trichrome (M-T) stain experiments were performed on the histological section, the stained samples were stuck on H&E, M-T paraffin plates. Hemocytometer microscopic images were used to observe the stain samples. The amount of collagen formation was determined by using the ImageJ RGB deconvolution technique.

**
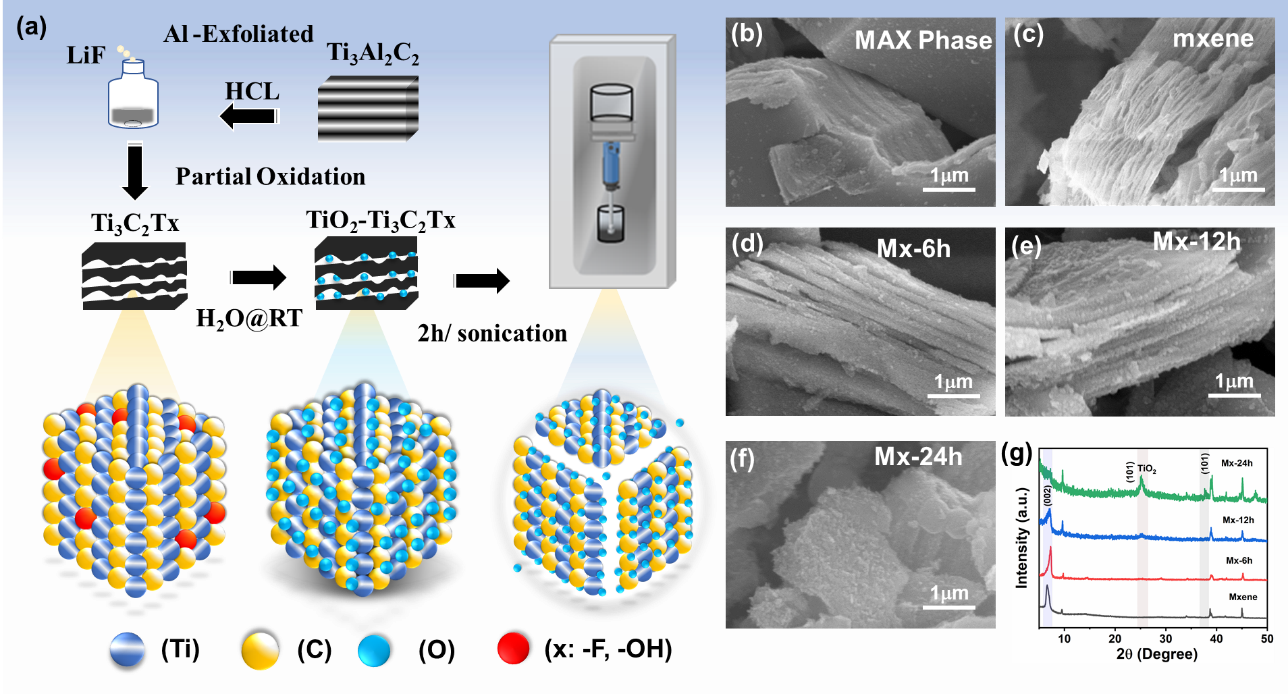
**Figure S1. Synthesis of oxidized MXene, (b-f) FE-SEM of oxidized MXene at different time intervals, (g) XRD spectrum

A systematic synthesis procedure of the TiO_2_/MXene preparation technique is illustrated in Figure S1. Step I, the MXene was derived by selective Al etching from MAX phase MXene. In the step II process, the controlled in-situ oxidation of MXene was performed to construct TiO_2_- MXene in an aqueous solution at room temperature. In step III, after drying 3 wt% was mixed in ethanol solution and placed under probe sonication to reduce the size of sheets. FE-SEM morphological structure of each step MXene is shown in Figure S1 (b-f). FE-SEM of synthesized MXene shows layer-by-layer morphology in Figure S1 (c), where the Al layers are etched from the MAX phase bulk sample (Figure S1 (b)). As described in materials and methods the oxidation stages (Low, Medium, and High) are differentiated by the oxidation time of 6, 12, and 24h). The medium oxidized TiO_2_-Ti_3_C_2_ was shown in Figure 1(b), here the superficial Ti atoms of MXene were oxidized into TiO_2_ nanoparticles. During, the oxidation process a metal-carbon bond cleaves and Ti-O bonds are formed (Figure. S1 (f)). Color of the MXene decolorized (become anatase) into ash color indicates the decomposition. Therefore, the controlled oxidation process becomes challenging in this process.


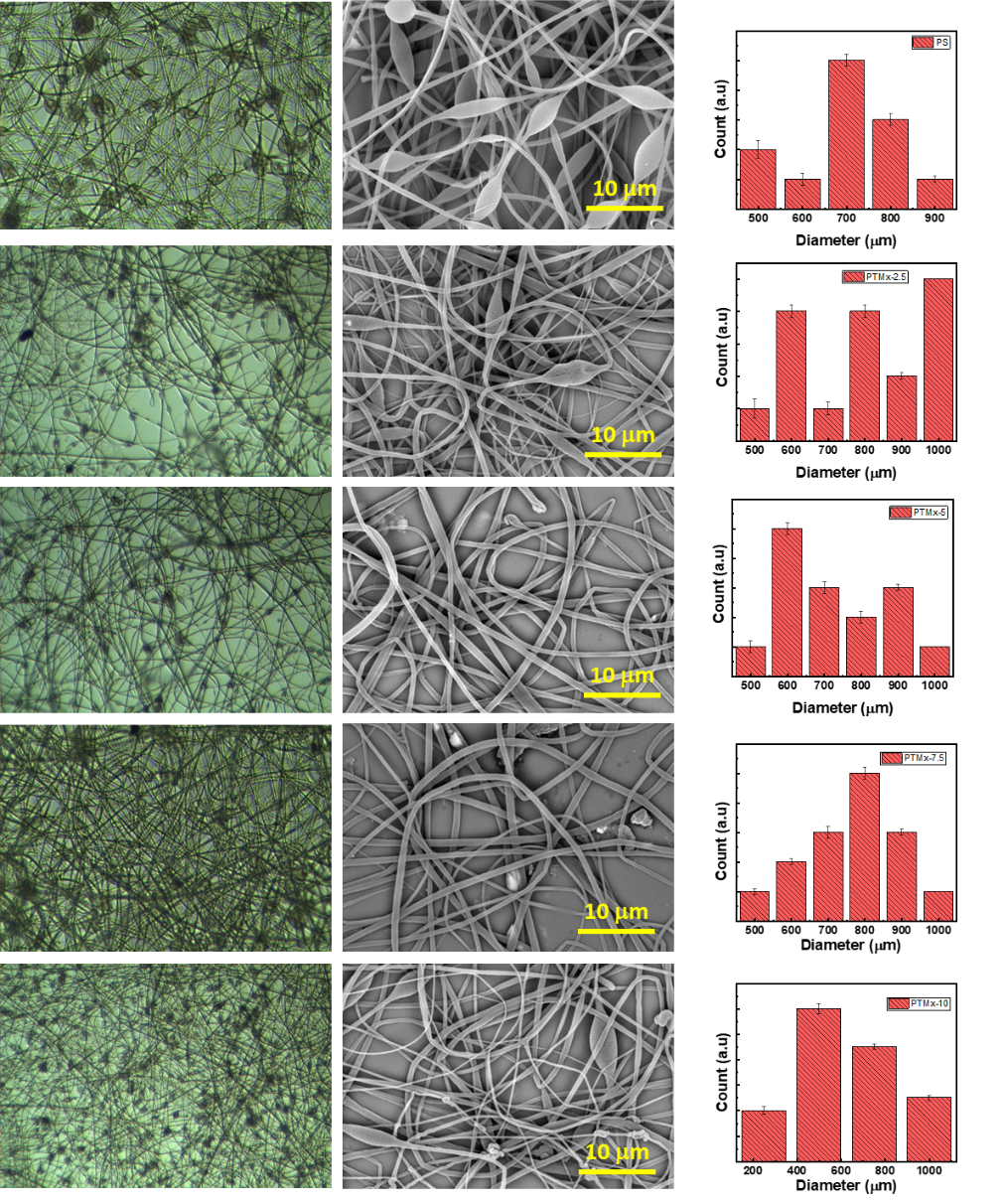


Figure S2. Optical microscopic image, SEM image of PS NF with 2.5, 5, 7.5, 10 wt% doping amount with diameter bar graph.


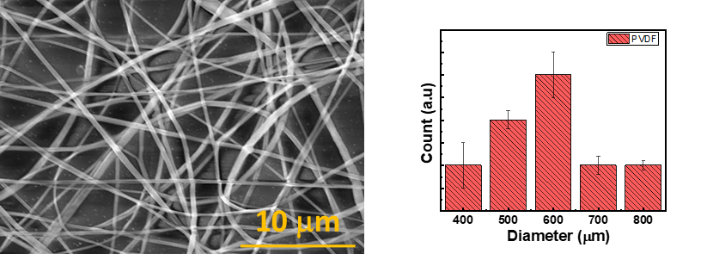


Figure S3. FE-SEM morphology of PVDF (control sample).





Figure S4. FTIR spectra of PVDF and PVDF/PU (PPU) NFM.

The absorption peaks at 761 cm^−1^ and 840 cm^−1^ are the characteristic peaks of PVDF α and b-crystal polymorphs. Addition of PU to PVDF increase the β crystallinity and suppressing α phase by making [TTTT] regular Trans configuration. The crystalline phase shows more tribo-negativity than the amorphous phase. The blue lines in the FTIR indicated the infrared spectra of the PU mixed PVDF NFM. The intense absorption peak at 1535 cm^−1^ indicated the N–H stretching vibration, and the band at 1725 cm^−1^ was the C=O stretching vibration ^[3][4]^.


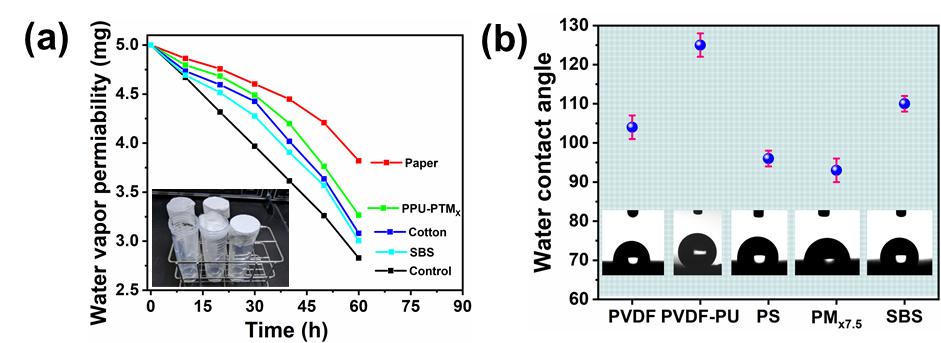


Figure S5. (a) water vapor permeation test (b) water contact angle test.

Table S1. Mechanical properties of PVDF and PPU NFM

|  | PVDF | PPU |
| --- | --- | --- |
| Young Modulus MPa | 1.07 | 1.19 |
| Elongation breaking point (%) | 23.21 | 73.71 |


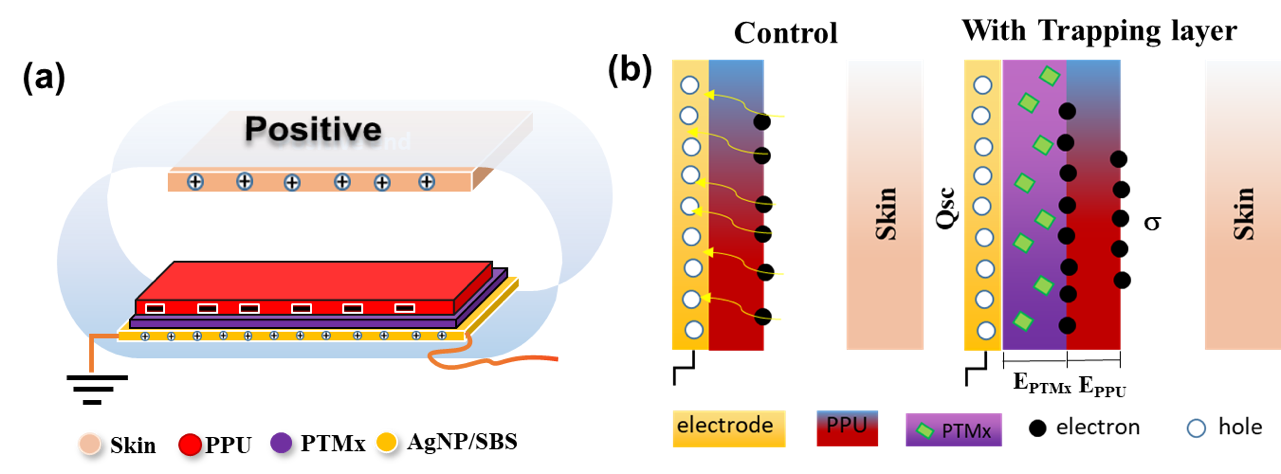


Figure S6. S-TENG device architecture (b) Charge transfer between a metal and a dielectric layer in contact electrification.

Schematically displays the S-TENG device fabrication and corresponding mechanism of tribo-electrification and electro-static potential induction between a metal and dielectric. When the skin contacts the dielectric surface, the electric static charges are induced on the PPU dielectric layer due to the difference in the work functions of the contact materials. Consequently, these electrostatic induction generate positive charges on metal electrodes such charge polarization creates a strong electric field. Then induced electric field disappears owing to the charge recombination with the electrode and atmospheric ions. Whereas, by placing the PTMx charge tapping interlayer the induced charges are stored at the metal-dielectric interface, which prevents charge neutralization effectively.


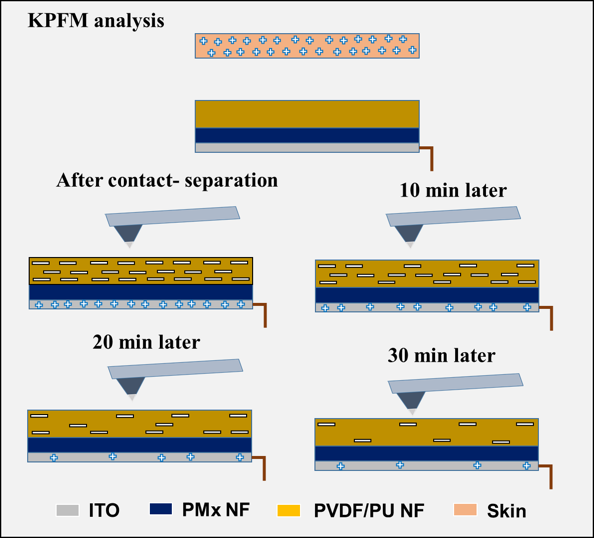


Figure S7. KPFM surface charge density measurement with various time intervals.


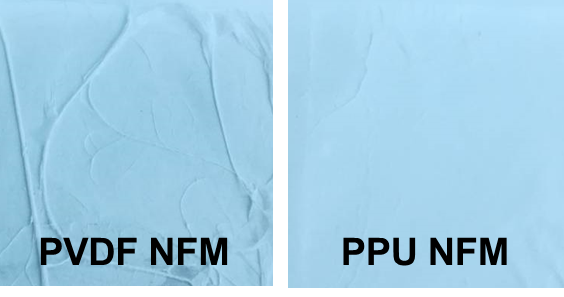


Figure S8. Surface morphology of PVDF and PPU surface friction layer after long cycle test.


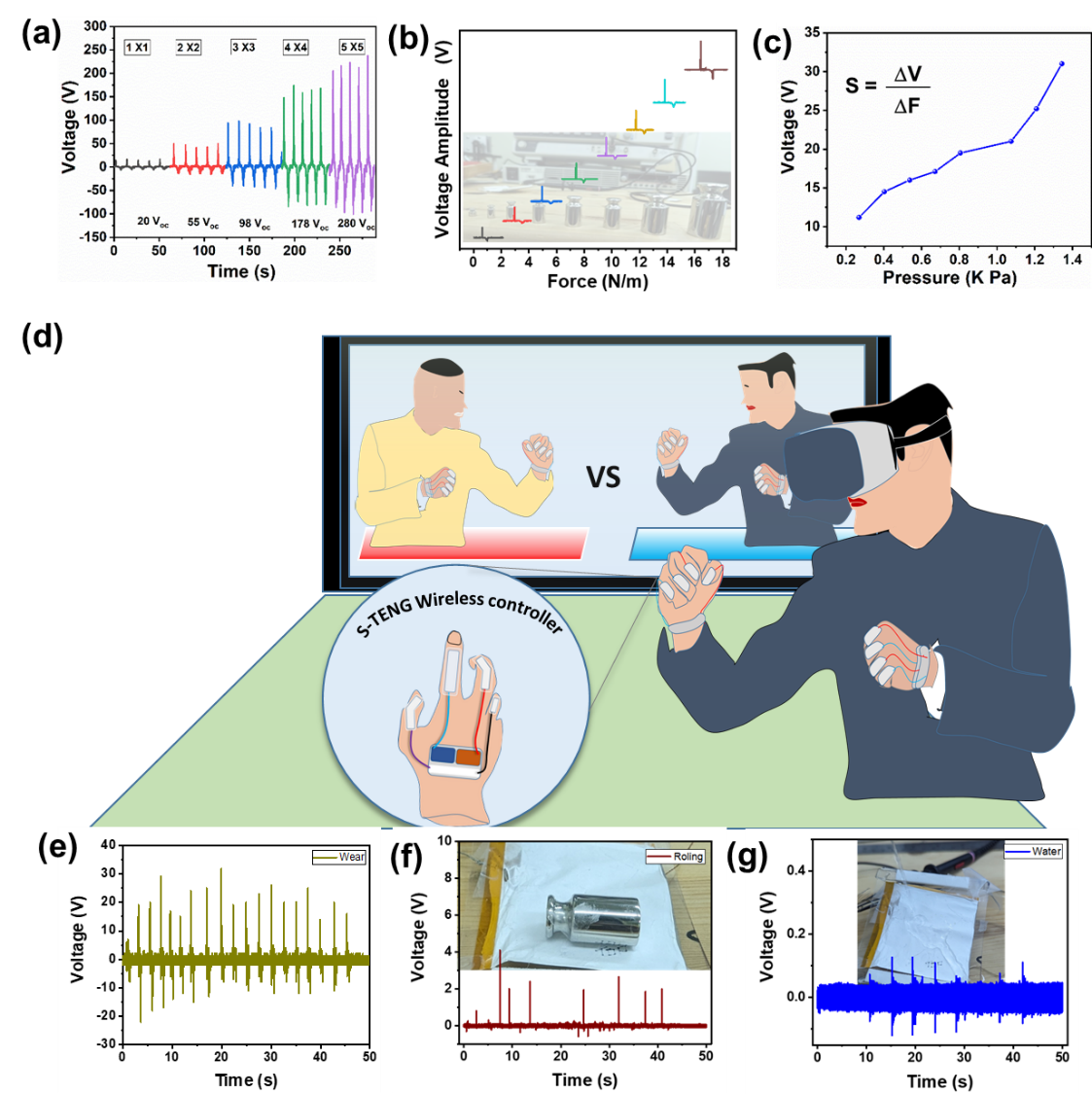


Figure S9. (a-b) shows the open circuit voltage of S-TENG with various areas and various loads (c) sensitivity of the device (d) graphical illustration of the wireless sensing (e) worn on cloth (f) Rolling friction (g) water dropping.

**Sensitivity calculation:**

An independently falling object depends on the physical model which consists of the gravity term and pulse term. Here the potential energy of the object is equal to the kinetic energy (I). Therefore, when the object falls from the known distance (A) to the surface of the film (B) the force is completely transferred on it, and the velocity becomes zero ^[4]^.

(SI)


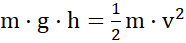

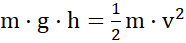


(SII)


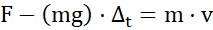

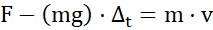


(SIII)


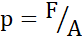

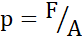


Here, m is the mass of the falling object is 0.005 kg, h is 0.1 m the distance between A and B. g is the gravity, v is the falling velocity of the object, F is the measured force, is the time span, p is the applied pressure, and A is the contact area of the film. The contact area (A) is 625 mm^2^, h = 0.1 m and g is 9.8 N/kg. Substituting these values in the above equations we found the contact force of the object is (F) approximately 0.42 N.


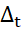

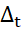


Sensitivity of the device was calculated using the following equation;

Sensitivity $=\frac{\Delta V}{\Delta F}$ (SIV)


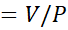


$\Delta V$ represents the open circuit voltage of applied pressure ($\Delta F$)


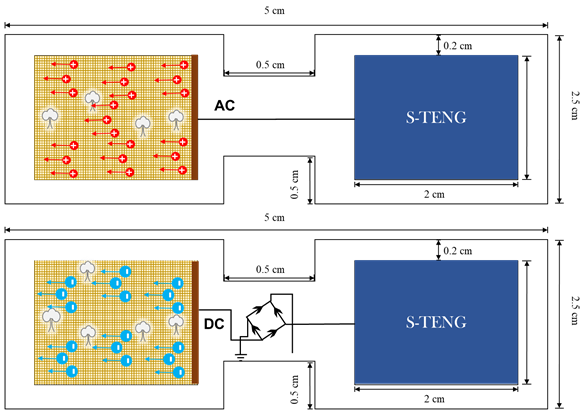


Figure S10. Device with AC and DC construction for wound healing

Video 1. Tensile property of PPU NFM

Video 2. Powering Red, Green, Blue LED lights and Timepiece

Video. 3 Smart keys demonstration with Snakebite game

Video 4. In vitro experiment

Video 5. Wound healing monitor

**Reference**

[1] R. A. Soomro, J. Kumar, R. R. Neiber, Sirajuddin, A. M. Alotaibi, S. F. Shaikh, N. Ahmed, A. Nafady, *Anal. Chim. Acta* **2023**, *1251*, 341016.

[2] S. Li, W.-C. Liu, Y.-H. Chang, X. Liu, C.-L. Chang, C. Lin, R.-J. Chung, *Mater. Sci. Eng. C* **2019**, *101*, 640.

[3] H. Dong, K. Xiao, X. Tang, Z. Zhang, J. Dai, R. Long, W. Liao, *Desalin. Water Treat.* **2016**, *57*, 3405.

[4] M. Venkatesan, W.-C. Chen, C.-J. Cho, L. Veeramuthu, L.-G. Chen, K.-Y. Li, M.-L. Tsai, Y.-C. Lai, W.-Y. Lee, W.-C. Chen, C.-C. Kuo, *Chem. Eng. J.* **2022**, *433*, 133620.
